# Effect of obesity on perioperative outcomes following gastrointestinal surgery: meta-analysis

**DOI:** 10.1093/bjsopen/zrad026

**Published:** 2023-07-10

**Authors:** Carolyn Cullinane, Anna Fullard, Stefanie M Croghan, Jessie A Elliott, Christina A Fleming

**Affiliations:** Department of Colorectal Surgery, University Hospital Waterford, Waterford, Ireland; Department of General and Colorectal Surgery, University of Limerick Hospital Group, Limerick, Ireland; Department of Urology, Royal College of Surgeons Ireland, St Stephen’s Green, Dublin, Ireland; Department of Surgery, Trinity St. James’s Cancer Institute, Trinity College Dublin, and St. James’s Hospital, Dublin, Ireland; Department of General and Colorectal Surgery, University of Limerick Hospital Group, Limerick, Ireland; Progress Women in Surgery Fellowship, Royal College of Surgeons in Ireland, Dublin, Ireland

## Abstract

**Background:**

Obesity can pose perioperative challenges related to obesity-associated co-morbidities and technical factors. However, the true impact of obesity on postoperative outcomes is not well established and reports are conflicting. The aim was to perform a systematic review and meta-analysis to explore the effect of obesity on perioperative outcomes for general surgery procedures in distinct obesity subtypes.

**Methods:**

A systematic review was performed for studies reporting postoperative outcomes in relation to BMI in upper gastrointestinal, hepatobiliary and colorectal based on an electronic search using the Cochrane Library, Science Direct, PubMed and Embase up to January 2022. The primary outcome was the incidence of 30-day postoperative mortality among patients with obesity undergoing general surgical procedures in comparison to patients with normal range BMI.

**Results:**

Sixty-two studies, including 1 886 326 patients, were eligible for inclusion. Overall, patients with obesity (including class I/II/II) had lower 30-day mortality rates in comparison to patients with a normal BMI (odds ratio (OR) 0.75, 95 per cent c.i. 0.66 to 0.86, *P* < 0.0001, *I*^2^ = 71 per cent); this was also observed specifically in emergency general surgery (OR 0.83, 95 per cent c.i. 0.79 to 0.87, *P* < 0.0000001, *I*^2^ = 7 per cent). Compared with normal BMI, obesity was positively associated with an increased risk of 30-day postoperative morbidity (OR 1.11, 95 per cent c.i. 1.04 to 1.19, *P* = 0.002, *I*^2^ = 85 per cent). However, there was no significant difference in postoperative morbidity rates between the cohorts of patients with a normal BMI and class I/II obesity (OR 0.98, 95 per cent c.i. 0.92 to 1.04, *P* = 0.542, *I*^2^ = 92 per cent). Overall, the cohort with obesity had a higher rate of postoperative wound infections compared with the non-obese group (OR 1.40, 95 per cent c.i. 1.24 to 1.59, *P* < 0.0001, *I*^2^ = 82 per cent).

**Conclusion:**

These data suggest a possible ‘obesity paradox’ and challenge the assumption that patients with obesity have higher postoperative mortality compared with patients with normal range BMI. Increased BMI alone is not associated with increased perioperative mortality in general surgery, highlighting the importance of more accurate body composition assessment, such as computed tomography anthropometrics, to support perioperative risk stratification and decision-making.

**Registration number:**

CRD42022337442 (PROSPERO https://www.crd.york.ac.uk/prospero/).

## Teaser

Results from this meta-analysis of 62 studies suggest a possible ‘obesity paradox’ and challenges the assumption that patients with obesity have higher postoperative mortality compared with patients with normal range BMI. Increased BMI alone is not associated with increased perioperative mortality in gastrointestinal surgery, highlighting the importance of more accurate body composition assessment, such as computed tomography anthropometrics, to support perioperative risk stratification and decision-making.

## Introduction

Obesity is a consequence of complex interactions between genetic, socioeconomic and cultural influences. In the last three decades, the worldwide prevalence of obesity has increased three-fold. In 2016, over 39 per cent of adults aged 18 years and older were overweight and 13 per cent were obese^1^. In Europe, obesity (BMI greater than or equal to 30 kg/m^2^) has reached epidemic proportions with the prevalence in men ranging from 4.0 to 28.3 per cent and in women from 6.2 to 36.5 per cent, with considerable geographical variation^2^. The most widely adopted classification of obesity in the Western world is the WHO criteria. A BMI of less than 18.5 kg/m^2^ is considered underweight and a BMI of 25–29.9 kg/m^2^ is considered overweight. The extent of obesity can then be further classified as: class I is specified for a BMI of 30–34.9 kg/m^2^, class II for a BMI of 35–39.9 kg/m^2^ and class III applies to those with a BMI greater than or equal to 40 kg/m^2^^3^. However, this grading system is better suited to Europeans as geographical differences in physiology and subsequently BMI are well documented. Asia-Pacific populations have different body fat distribution and therefore morbidity and mortality can occur in this population group with lower BMIs. To address this variability, in 2000 a BMI scale for Asian adults was proposed with a normal BMI range of 18.5–22.9 kg/m^2^ and obesity class I defined as a BMI of 25–29.9 kg/m^2^^4^.

Obesity is associated with several co-morbidities, including diabetes, hypertension, coronary artery disease and increased risk of certain cancers^3^. Every five-unit increase in BMI above 25 kg/m^2^ is believed to increase mortality rate by 30 per cent^5^. The obesity epidemic also impacts surgery and surgical outcomes, not merely because of an increase in the prevalence of obesity but also because of an increase in obesity-related surgical diseases^6^. Obesity can pose several perioperative challenges, including management of co-morbidities, as well as technical and equipment-related issues^7^. However, the true impact of obesity on postoperative outcomes is not well established and reports across a wide range of surgical studies are conflicting.

The obesity paradox, which suggests that patients with obesity have more favourable postoperative outcomes compared with those who have a normal BMI, was first conceptualized in relation to cardiac surgery in the early 2000s^8,9^. Since then, several conflicting studies have reported on the subject of the obesity paradox in general surgery. Mullen *et al.* prospectively examined patients undergoing non-bariatric general surgery and demonstrated that patients with obesity had a lower risk of mortality compared with patients who have a normal BMI^10^. These results have been replicated in larger cohort studies comparing general surgical mortality rates between patients with elevated BMI to a non-obese reference group^11–13^. More recently, obesity was reported to be particularly protective in older adults undergoing emergency surgery^14^. In contrast, Kassahun *et al*. found that patients with obesity were more likely to have co-morbidities and were at increased risk of postoperative complications and mortality following emergency laparotomy for high-risk abdominal emergencies^15^. Similarly, postoperative complication and wound infection rates have also been reported to be higher in general surgical patients with obesity in multi-institutional cohort studies^16,17^. The aim of this study was to review the existing literature with respect to the impact of obesity on perioperative outcomes among patients undergoing general surgery operations.

## Methods

**Fig. 1:**
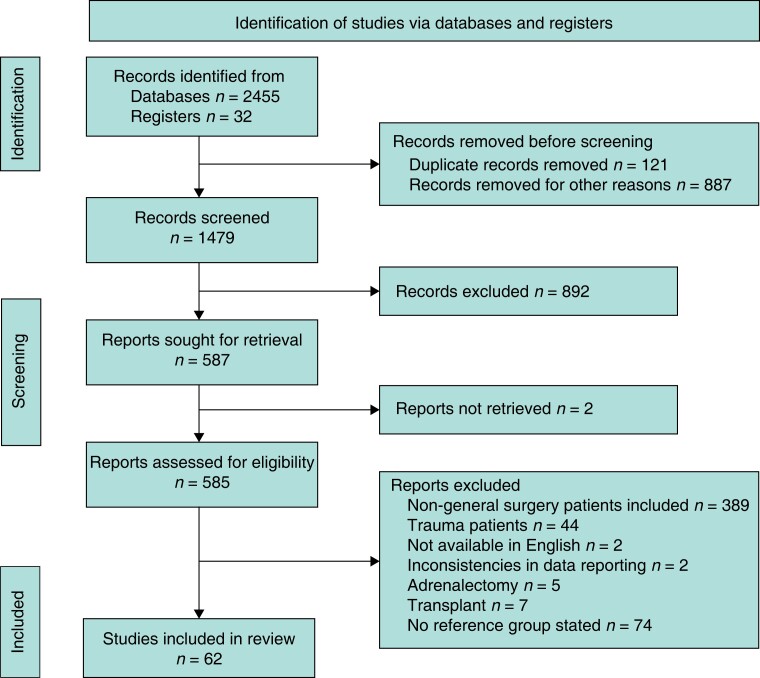
PRISMA flowchart

This study was performed following guidance from the PRISMA and Meta-Analysis of Observational Studies in Epidemiology (*Fig. 1*)^18^. Prospective registration was performed on PROSPERO (CRD42022337442).

### Search strategy

An electronic search was conducted using the Cochrane Library, Science Direct, PubMed and Embase. All studies published to January 2022 were included. The following search terms/MeSH terms were used: (Obesity (MeSH) OR obese OR Body Mass Index OR in high BMI OR elevated BMI OR BMI) AND (general surgery (MeSH) OR surgery OR non bariatric surgery OR colorectal surgery OR gastrointestinal surgery OR GI surgery OR abdominal surgery OR hepatobiliary surgery OR Hepatobiliary (HPB) surgery emergency surgery OR oesophageal surgery OR liver surgery OR gastric surgery) AND (postoperative outcomes (MeSH) OR complications OR outcomes). All titles were initially screened, and appropriate abstracts were reviewed by two independent reviewers (C.C., A.F.). Each publication bibliography and Google Scholar were manually searched for relevant articles. The last date of search was 31 January 2022.

### Outcomes

The primary outcome was the incidence of 30-day postoperative mortality among patients with obesity undergoing general surgical procedures in comparison to patients with normal range BMI. Obesity class subgroup categorization into class I, II and III obesity as per the WHO criteria was performed^1^. Asia-Pacific populations have different body fat distribution and have lower BMI cut-off values. Population-specific values were considered when subgrouping studies into the different BMI classes. The secondary outcomes were the incidence of in-hospital mortality, 30-day postoperative complications (classified as Clavien–Dindo >/= II) and surgical site infection (SSI) (classified as superficial or deep SSI). Clavien–Dindo >/= II morbidities were included to capture all complications requiring pharmacological or surgical intervention as these interventions impact the duration of hospital stay and postoperative patient experience. A subgroup analysis was also performed on studies including emergency general surgery patients. The quality of the studies included was assessed using the Newcastle–Ottawa Scale (NOS)^19^. The risk of bias was assessed using the Risk of Bias in Non-Randomized Studies of Intervention (ROBINS I Tool)^20^. Publication bias was assessed using visual inspection of funnel plots.

### Inclusion criteria

All studies (observational, non-randomized) meeting the following criteria were suitable for inclusion: studies that included elective and emergency general surgery including upper gastrointestinal (GI), hepatobiliary and colorectal; studies that included a small cohort of non-general surgery patients (that is urology, vascular) with general surgery patients; studies that included non-obese as a reference group and reported the number of patients in individual BMI categories with reported outcomes of interest.

### Exclusion criteria

The following exclusion criteria were applied: studies published in languages other than English; studies that focused on non-GI surgery patient groups, including organ transplant and adrenalectomy^21^; studies on trauma due to complexity of their multiple organ injury and physiology; studies did not present standardized incidence ratio, odds ratios, risk ratios or hazard ratio estimates (with 95 per cent c.i.), standard errors or number of events necessary to calculate these for the outcomes of interest.

### Data extraction

Two reviewers (C.C., A.F.) independently screened all titles/abstracts/texts for eligibility according to the above predefined strategy and criteria. Each reviewer extracted the following variables: title and study details (year, design, country), study population characteristics (sample size, subspecialty, elective *versus* emergency, obesity subtypes, outcomes of interest and number of events). In cases of disagreement, a consensus was reached by discussion and agreed with a third reviewer (C.F.).

### Statistical analysis

Statistical analysis was performed using Review Manager 5 (The Cochrane Collaboration, The Nordic Cochrane Centre, Copenhagen, Denmark). Binary outcome data were reported as odds ratios (OR) with 95 per cent c.i. using the Mantel–Haenszel method. Adjusted odds ratios (OR) reported in the study publication were used when available; otherwise, they were extrapolated from the available data. Weighted mean differences (MDs) were calculated for the effect size on continuous variables. Heterogeneity was assessed using *I*-squared statistics, with >10 per cent being considered significant heterogeneity. A fixed-effects model was preferred to a random-effects model when there was no heterogeneity, otherwise a random-effects model was used. Pooled estimates of differences were calculated using random-effects models, accounting for potential interstudy heterogeneity. *P* values of <0.05 were considered significant.

## Results

### Characteristics of included studies

As outlined in *Fig. 1*, 62 studies, including 1 886 326 patients, were eligible for inclusion^10–17,22–75^. All studies were published between 2001 and 2022. Eleven studies were prospectively designed^11,12,16,23,43,47,48,52,61,72,73^ and the remaining 51 studies were retrospective^10,13–15,17,22,24–30,32–42,44–46,49–51,53–60,62–67,74,75^. Two of the prospectively designed studies were matched case-controlled^43,47^ and eight retrospective studies included matched controls^28,33,42,44–46,49,54^.

Countries of origin included: USA (*n* = 27), the Netherlands (*n* = 4), France (*n* = 4), Japan (*n* = 6), Germany (*n* = 5), Switzerland (*n* = 2), Denmark (*n* = 1), Chile (*n* = 1), South Korea (*n* = 1), Ireland (*n* = 2), Canada (*n* = 1), Australia (*n* = 1), Czech Republic (*n* = 1), UK (*n* = 2), Turkey (*n* = 1), Spain (*n* = 1) and China (*n* = 1). The breakdown of the different subspecialties of general surgery was also diverse: emergency general surgery (*n* = 5), colorectal (*n* = 34), elective general surgery (*n* = 9), upper GI (*n* = 12) and hepatobiliary (*n* = 2). Of these studies, three involved robotic surgery (colorectal (*n* = 2) and upper GI (*n* = 1)). Sixty-seven per cent (*n* = 42) of the included studies had a NOS of 8 or 9. The remaining 21 studies had a score of 6 or 7 (*Table 1*).

**Table 1:** Characteristics of included studies (*n* = 62)

	*n* (%)
**Year of publication**	
2000–2006	9 (15)
2007–2012	22 (36)
2013–2017	19 (31)
2018–2022	12 (18)
**Median (range) age (years)**	66.6 (17–91)
**Country of origin**	
North America	28 (46)
South America	1 (2)
Asia	8 (12)
Europe	23 (38)
Australia	1 (2)
**Study design**	
Prospective	11 (18)
Retrospective	51 (82)
**Subspecialty**	
Emergency general surgery (laparotomy, duodenal/bowel perforation, bowel obstruction)	5 (8)
Elective general surgery (abdominal oncological resections, hernia repair, retroperitoneal dissection, liver surgery, cholecystectomy)	9 (15)
Colorectal (colorectal resections, pouch surgery, rectal prolapse surgery)	34 (56)
Upper gastrointestinal (gastrectomy, oesophagectomy)	12 (18)
Hepatobiliary (liver resection, pancreato-duodenectomy)	2 (3)
**Newcastle-Ottawa score**	
8–9	42 (67)
6–7	21 (33)

Values are *n* (%) unless otherwise stated.

### 30-day morbidity

**Fig. 2:**
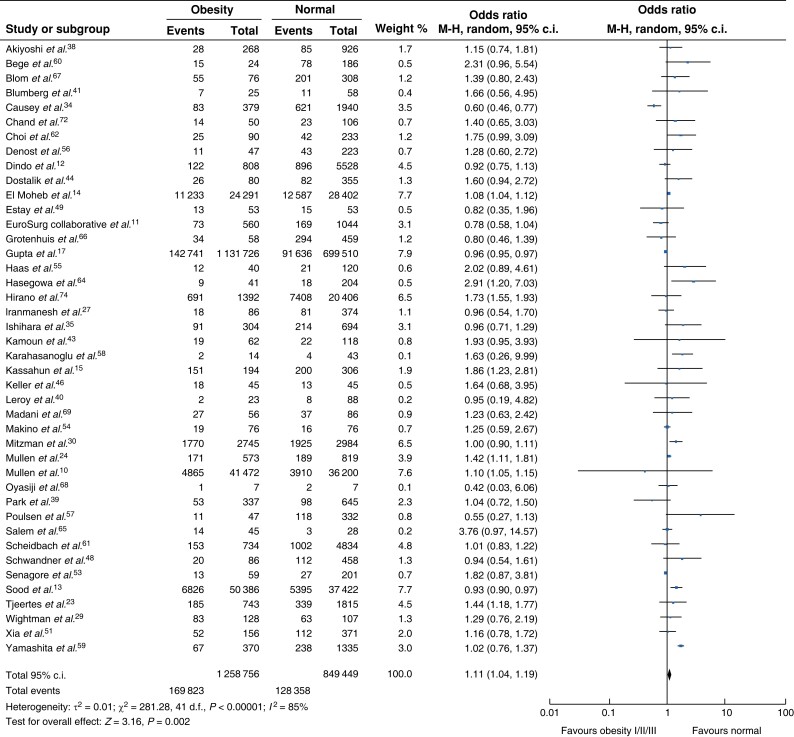
**Thirty-day morbidity rate between non-obese patients and patients with obesity** M-H, Mantel-Haenszel method.

Forty-two studies (*Fig. 2*) were eligible for inclusion^11–17,23,26,35,37–39,42,43,45–47,50–52,54–56,58–60,62–66,70–74^. Compared with a normal BMI, obesity was positively associated with an increased risk of 30-day postoperative morbidity, although significant heterogeneity between studies was observed (OR 1.11, 95 per cent c.i. 1.04 to 1.19, *P* = 0.008, *I*^2^ = 79 per cent). Comparing normal BMI to class I obesity^10,12,14,17,24,27,29,30,61^ or class I/II obesity combined^10,13,14,17,24,30^, no significant difference in 30-day morbidity was observed (*Fig. S1*). More favourable outcomes for patients with a normal BMI compared with class II/III obesity combined were observed^10,12,14,17,24,27,29,30,61^ (OR 1.14, 95 per cent c.i. 1.02 to 1.27, *P* = 0.022) and results demonstrated significant heterogeneity (*I*^2^ = 93 per cent).

### Surgical site infection

**Fig. 3:**
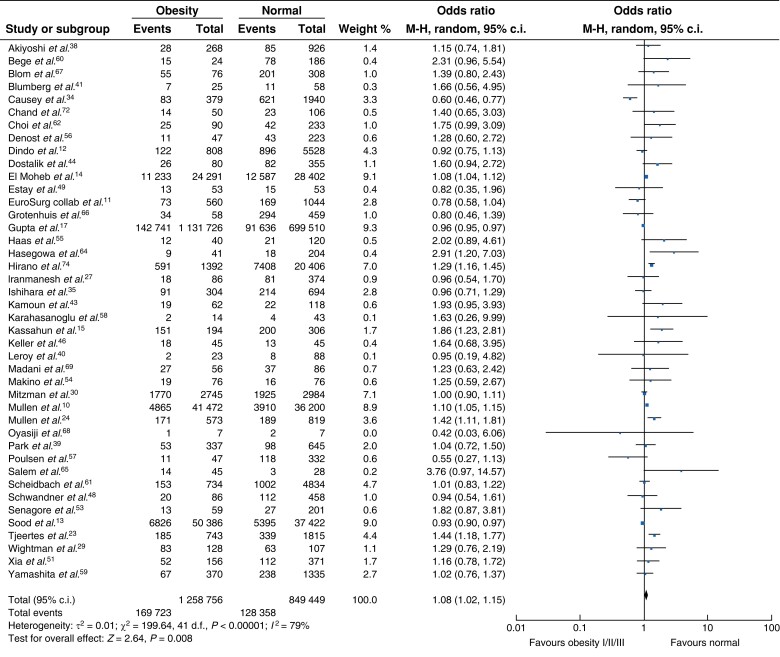
**Surgical site infection among patients with normal BMI compared with obesity** M-H, Mantel-Haenszel method.

**Fig. 4:**
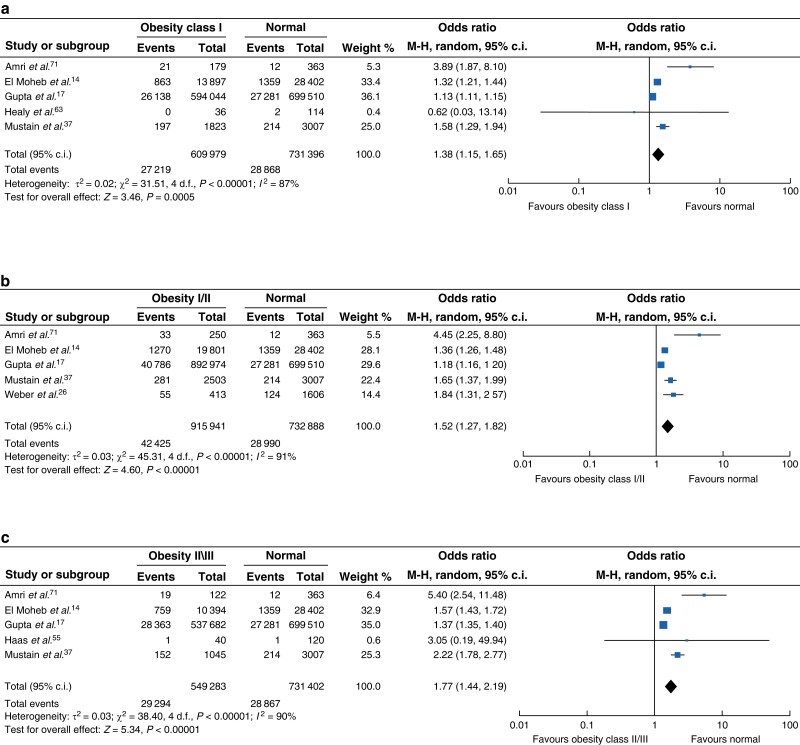
**Surgical site infection (SSI) among patients with normal BMI compared with obesity sub-class a** SSI among patients with BMI as compared with class I obesity; **b** SSI among patients with normal BMI as compared with class I/II obesity; **c** SSI among patients with normal BMI as compared with class II/III obesity. M-H, Mantel-Haenszel method.

Thirty-six studies reported the incidence of SSI between patient cohorts with and without obesity^12–17,23,26,35,37–39,42,43,45–47,50–52,54–56,58–60,62–66,70–73,75^. Overall, the non-obese cohort had a statistically lower rate of SSI compared with patients with obesity (*Fig. 3*), although data were heterogenous (OR 1.40, 95 per cent c.i. 1.24 to 1.59, *P* < 0.001, *I*^2^ = 82 per cent). Five studies compared SSI rates between patients with normal range BMI and patients with class I obesity^14,17,37,63,71^ and demonstrated a significantly higher rate of SSI (*Fig. 4a*, OR 1.38, 95 per cent c.i. 1.15 to 1.65, *P* < 0.005). Compared with patients with a normal range BMI, patients with class I/II obesity had a significantly higher rate of SSI (*Fig. 4b*, OR 1.52, 95 per cent c.i. 1.27 to 1.82, *P* < 0.001)^14,17,26,37,71^. SSI rates among patients with class II/III obesity were also significantly higher compared with those with a normal BMI, however, there was significant heterogeneity between studies^14,17,37,55,71^ (*Fig. 4c*, OR 1.77, 95 per cent c.i. 1.44 to 2.19, *P* < 0.001, *I*^2^ = 90 per cent).

### Thirty-day mortality and in-hospital mortality

**Fig. 5:**
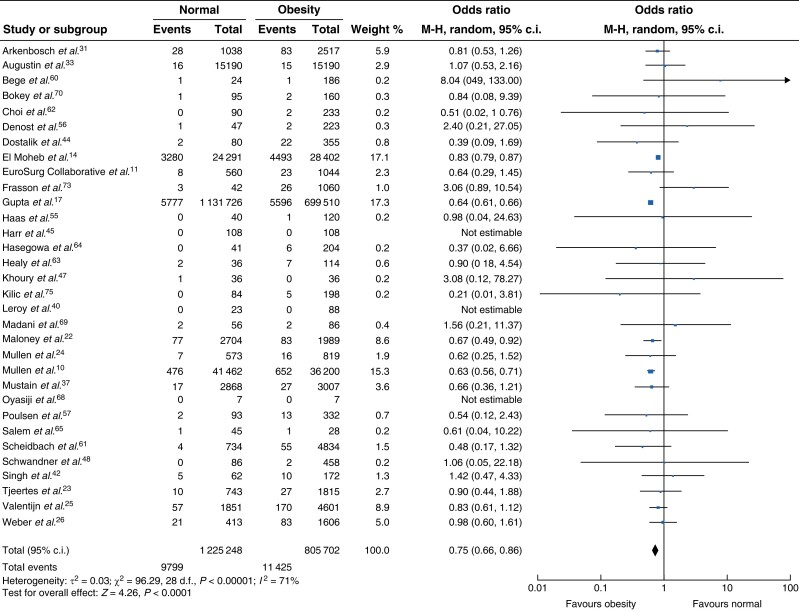
**Thirty-day mortality among patients with normal BMI compared with obesity** M-H, Mantel-Haenszel method.

Thirty-two studies reported 30-day mortality rates between non-obese and obese patient cohorts^10,11,14,17,22–26,31,33,37,40,42,44,45,47,48,55–57,60–65,68–70,73,75^. Overall, patients with obesity appeared to have significantly lower 30-day mortality rates in comparison to patients with a normal BMI (OR 0.75, 95 per cent c.i. 0.66 to 0.86, *P* < 0.0001). However, there was significant heterogeneity between the studies *I*^2^ = 71 per cent (*Fig. 5*). Nine studies compared mortality in normal BMI and class I obesity^10,14,17,22,24,25,37,61,63^. Patients with class I obesity had lower mortality in comparison to normal weight patients (OR 0.69 *P* < 0.001) with significant heterogeneity between the studies *I*^2^ = 83 per cent (*Fig. S2a*). In the eight studies comparing normal BMI and class I/II obesity combined^10,14,17,22–25,37^, patients with a normal BMI demonstrated a higher 30-day mortality rate in comparison to patients with class I and/or II obesity (*Fig. S2b*, OR 0.78, 95 per cent c.i. 0.64 to 0.93, *P* = 0.007) with significant heterogeneity between the studies (*I*^2^ = 94 per cent). This was also demonstrated in the comparison of normal BMI and class II/III obesity combined^10,14,17,22,24–26,33,37,61^, again favouring higher BMI (*Fig. S2c*, OR 0.78, 95 per cent c.i. 0.65 to 0.93, *P* = 0.006, *I*^2^ = 87 per cent). Fifteen studies compared the in-hospital mortality between patients with a normal BMI and all classes of obesity combined^12,13,15,22,28–30,32,35,36,43,64,66,67,71,74^. Comparisons according to the class of obesity were not possible due to limited reporting of obesity classes. Overall, there was no difference in in-hospital mortality between patients with obesity and patients without obesity (*Fig. S3*, OR 1.15, 95 per cent c.i. 0.79 to 1.67, *P* = 0.462, *I*^2^ = 90 per cent).

### Emergency general surgery

**Fig. 6:**
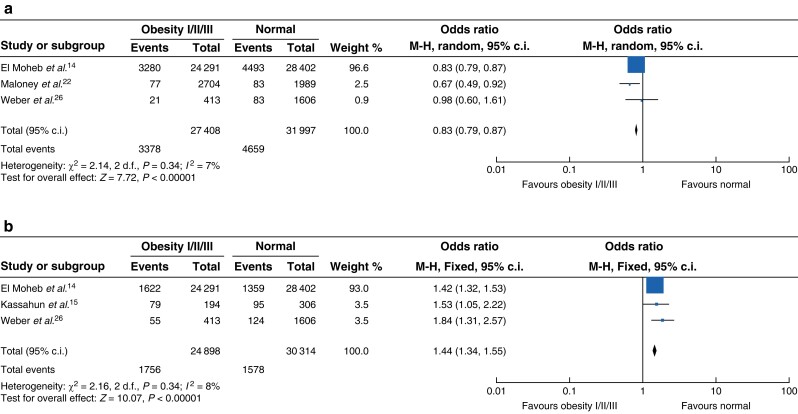
**Outcomes in emergency general surgery comparing normal BMI and obesity (all classes)** **a** Thirty-day mortality following emergency general surgery among patients with a normal BMI as compared with obesity. **b** Surgical site infections following emergency general surgery among patients with a normal BMI as compared with obesity. M-H, Mantel-Haenszel method.

Five studies reported on emergency general surgery patients only^14,15,22,26,32^. Three studies reported 30-day mortality in emergency general surgery patients with a normal BMI compared with patients with obesity^14,22,26^. Patients with an elevated BMI had a significantly lower 30-day mortality rate (*Fig. 6a*, OR 0.83, 95 per cent c.i. 0.79 to 0.87, *P* < 0.001, *I*^2^ = 7 per cent). Subgroup analysis of three suitable studies reporting SSI rates^14,15,26^ showed that patients with obesity had higher rates of wound infection (OR 1.44, 95 per cent c.i. 1.34 to 1.55, *P* < 0.001) with little heterogeneity between the studies (*I*^2^ = 8 per cent) (*Fig. 6b*).

## Discussion

This systematic review and meta-analysis investigated the effect of obesity on postoperative morbidity and mortality following GI surgery. Interestingly, no difference in 30-day morbidity between patients with a normal BMI and obesity was demonstrated, when obesity classes were considered individually. However, when all obesity classes were combined, there was an increased risk of 30-day postoperative morbidity among patients with obesity compared with patients with a normal BMI. Conversely, the 30-day postoperative mortality rate was significantly lower in patients with obesity compared with patients with a normal BMI.

The existence of an ‘obesity paradox’ relative to short-term 30-day survival outcomes among patients undergoing non-bariatric surgery was previously described by Mullen *et al*.^10^. Their large, prospective, multi-institutional study of 118 707 patients undergoing non-bariatric surgery demonstrated a reverse J-shaped relationship between BMI and 30-day mortality with the highest event rate observed among those who were underweight and those with severe obesity, and the lowest rates among those who were classed as overweight and moderately obese^10^. These results concurred with a single institution study of 6336 patients from Switzerland examining the impact of obesity on elective general surgery outcomes. Obesity was reported to be neither protective of or a risk factor for death or complications in patients undergoing elective surgery^12^. The long-term validation of the ‘obesity paradox’ was further reported by two general surgery studies with median follow-up times of over 5 years^23,25^. The concept that obesity incurs a reduced risk perioperatively is counterintuitive given that obesity is associated with various co-morbidities, including type 2 diabetes mellitus, hypertension, cerebrovascular events, coronary artery disease and an increased risk of death^76,77^.

Several biological mechanisms have been proposed to explain this obesity paradox. One such theory relates to the synergistic relationship between metabolomics and the immune response^78^. Immunological response to trauma or surgical insult initiates an acute phase inflammatory response^79^. Patients with obesity exhibit chronic, low-grade inflammation at baseline^80^. This chronically activated ‘meta-inflammatory’ state is the same signalling response required by the hosts’ immune response to respond appropriately to surgical trauma and initiate tissue healing^81^, suggesting that patients with obesity are primed immunologically to deal with the surgical insult. Another proposed theory on the protective effect of obesity on perioperative outcomes centres on the nutritional reserve available to mount the appropriate stress response to injury. This only applies to the obese and moderately obese cohorts as patients with severe obesity are believed to be ineffective in their energy use resulting in hyperbolic inflammatory responses, oxidative stress and immunosuppression^10^. The concept that patients with obesity have an increased metabolic reserve was analysed in a cohort of patients with obesity admitted to ICU following trauma where the authors demonstrated that in obesity, abundant fat stores are not effectively used as a fuel source and there is greater reliance on other fuel sources, such as endogenous protein^82^.

Findings from this meta-analysis suggest that when all classes of obesity are grouped together and compared with patients with a normal BMI, there is a slight increased risk in 30-day postoperative mortality in the non-obese group. A recent study demonstrated that in-patient mortality in patients undergoing emergency laparotomy varied according to weight classification, with patients with a BMI >40 having the worst outcome^15^. However, following adjustment for specific co-morbidities, BMI itself was not found to be an independent factor predictive of in-hospital mortality^15^. Similarly, Yanquez *et al*. suggested that increasing age combined with higher BMI was positively associated with morbidity and mortality, however, BMI itself was not an independent factor predicting 30-day complications^83^. A previous meta-analysis of 10 observational studies examined the perioperative outcomes of rectal cancer surgery in obese and non-obese cohorts and concluded that obesity increases the conversion rate (from laparoscopy to open) and postoperative morbidity of rectal cancer surgery but does not influence pathological results. However, six of the studies included underweight and overweight patients in the non-obese reference group, which may limit the generalizability of the results^84^. Similarly, a recent multicentre Japanese study observed that patients with a BMI >30 have an increased risk of postoperative complications following laparoscopic colorectal surgery (OR 2.6)^59^. Furthermore, a multicentre collaborative study conducted in the UK and Ireland suggested that overweight and obese patients undergoing surgery for GI malignancy, but not benign disease, were at increased risk of major postoperative complications compared with those of normal weight. These findings were explained by the fact that those undergoing surgery for benign conditions are likely to be subject to a selection bias of fitter patients for generally lower-risk procedures^16^. It was not possible to examine patients with benign and malignant conditions in this meta-analysis due to the heterogeneity of included studies, however, a follow-up European collaborative study (EuroSurg Collaborative) concluded that obesity was not found to be associated with major complications following GI surgery^11^.

Subgroup analysis of 36 eligible studies in the present study indicated that SSI rates were significantly lower in patients with a normal BMI (OR 0.71). The positive association between SSI and increased BMI is an observation that is echoed throughout the literature^23,26,59^. Wound infection rates in patients with obesity could be attributed to excess adipose tissue, which has low regional oxygen tension and is therefore susceptible to impaired wound healing and infection^12^. Furthermore, immune dysregulation and chronic cytokine secretion associated with obesity results in an immunosuppressive state, which likely contributes to higher rates of wound infection^80^. The tension on suture lines might be stressed in patients with obesity due to increased subcutaneous fat tissue, which would explain why laparoscopic surgery is advantageous in reducing SSI compared with open surgery in elective general surgery^12,85^.

This meta-analysis of 1 846 920 patients challenges the assumption that patients with obesity have a higher incidence and severity of major complications perioperatively compared with non-obese patients. Obesity defined by BMI does not appear to be a major risk factor in GI surgery. Given that body fat increases and muscle mass decreases with age, BMI may not accurately reflect changes in body fat or muscle mass and does not provide data on body fat distribution^86^. Abdominal obesity characterized by visceral fat accumulation measured using computed tomography (CT) may be a more accurate predictor of metabolic dysregulation in obesity^86^. Furthermore, BMI does not correctly capture the subset of patients with ‘metabolically healthy’ obesity, who do not experience the expected metabolic complications of obesity, most likely due to less visceral fat and preserved insulin sensitivity^87,88^. Conversely, patients with a normal BMI could be harbouring a disproportionately high mortality risk due to central obesity characterized by excessive visceral fat^89^.

There has been a shift in interest towards using more precise measures of body composition such as CT-derived abdominal measurement to accurately predict postsurgical outcomes. Kuritzkes *et al*. used CT-derived anthropometric results to predict postoperative morbidity in 264 patients undergoing colon resection for cancer and revealed that visceral fat accumulation, not BMI, predicts morbidity following elective surgery for colon cancer^90^. The clinical validity of visceral fat accumulation in predicting postoperative complications was corroborated by several other studies^91,92^. More recent studies indicate that the visceral to subcutaneous fat ratio and visceral to total fat ratio may be a more accurate predictive marker of postoperative outcomes because these values capture adipose tissue distribution and the technical challenges associated with central adiposity^93,94^. Fleming *et al*. reported that subcutaneous fat, which is considered relatively benign, was associated with higher levels of cytokines with anti-inflammatory properties, including interleukin 2 (IL-2) and IL-10, whereas patients with a high visceral to total fat ratio who developed recurrence had higher levels of the proinflammatory IL-6 and TNFα^93^. These findings suggest that different body composition profiles display their own unique inflammatory landscape, which may impact on postoperative outcomes.

The strength of this study lies in the large number of studies available for analysis spanning over two decades. Obesity subclassification according to BMI class also provides a more precise estimate of perioperative risk. However, there are several limitations to this study. The majority of the studies included were retrospective in nature and therefore subject to recall and information bias, particularly when relying on self-reported measurements and retrospective data from chart reviews. Similarly, there may be an element of selection bias present in the studies as patients with obesity may have been carefully selected for elective surgery because they displayed ‘healthy’ parameters. Subgroup analysis of emergency general surgery procedures was performed in an attempt to filter out patients who were carefully selected for elective surgery due to a healthier phenotype. As sarcopenia and an underweight BMI are consistently associated with poorer surgical outcomes in the literature, it is important to note that 21 of the studies included underweight patients in the non-obese comparator group^26,32,42–50,53,54,57,59,60,62,67,69,70,72^.

## Supplementary Material

zrad026_Supplementary_Data

## Data Availability

All data generated or analysed during this study are included in this manuscript (and its supplementary evidence). The data sets generated during the current study are available upon request from the corresponding author.
