# Supplementary material for: Effect of obesity on perioperative outcomes following gastrointestinal surgery: meta-analysis
Source: BJS Open. 2023 Jul 10;7(4):zrad026. doi: 10.1093/bjsopen/zrad026 (PMC10332403; doi:10.1093/bjsopen/zrad026)

**The Effect of Obesity on Perioperative Outcomes following Gastrointestinal Surgery: meta-analysis**

*C Cullinane ^1^, A Fullard ^2^, SM Croghan ^3^, JA Elliott ^4^, CA Fleming^2,5^*

*On behalf of the Irish Surgical Research Collaborative*

*Corresponding author: Carolyn Cullinane,* [*Carolyncullinane@rcsi.com*](mailto:Carolyncullinane@rcsi.com)*, 0000-0002-9320-1586*

*1. Department of Colorectal Surgery, University Hospital Waterford, Ireland*

*2. Department of General and Colorectal Surgery, University of Limerick Hospital Group, Ireland*

*3. Royal College of Surgeons Ireland, St Stephen’s Green, Dublin 2, Ireland*

*4. Trinity St. James’s Cancer Institute, Trinity College Dublin, and St. James’s Hospital,*

*Dublin, Ireland*

*5. PROGRESS Fellow, Royal College of Surgeons in Ireland, Dublin, Ireland*

**Supplementary Materials - Index**

| **Supplementary Figures** |  |
| --- | --- |
| **Figure S1: 30-day morbidity subgroup analysis by obesity class and compared to normal BMI** | *pag. 2* |
| **Figure S2: 30-day mortality subgroup analysis by obesity class and compared to normal BMI**  **Figure S3: In-hospital mortality among patients with normal BMI as compared with obesity (all classes)** | *pag. 3*  *pag.4* |
|  |  |

**Figure S1: 30-day morbidity subgroup analysis by obesity class and compared to normal BMI**


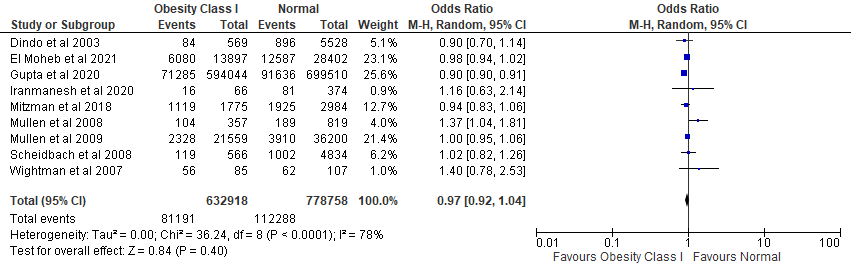


**A:** 30-day morbidity among patients with normal BMI as compared with class I obesity


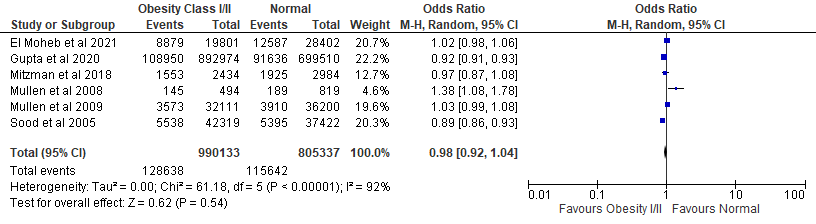


**B:** 30-day morbidity among patients with normal BMI as compared with class I/II obesity


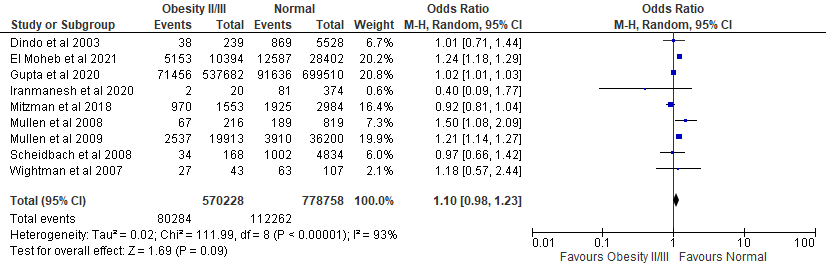


**C:** 30-day morbidity among patients with normal BMI as compared with class II/III obesity

**Figure S2: 30-day mortality subgroup analysis by obesity class and compared to normal BMI**


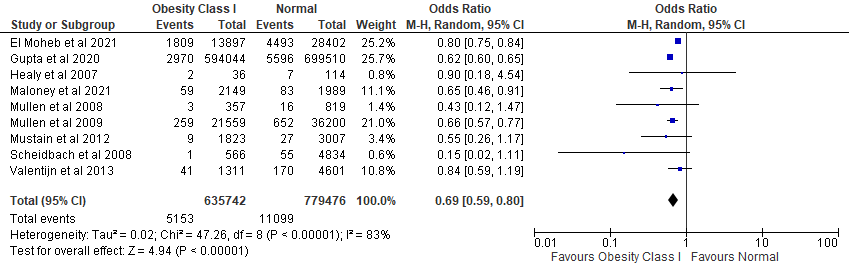


**A**: 30-day mortality among patients with normal BMI and Class I obesity


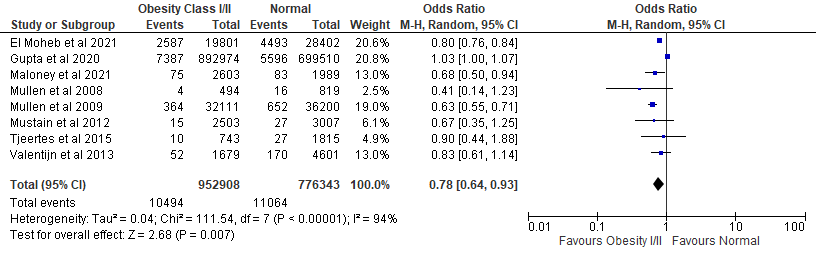


**B**: 30-day mortality among patients with normal BMI and Class I/II obesity

**
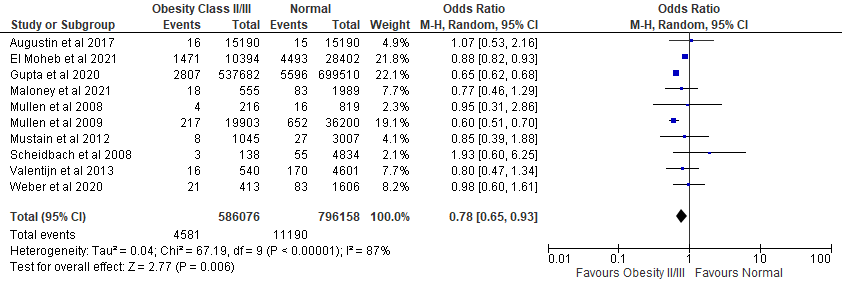
**

**C**: 30-day mortality among patients with normal BMI and those with Class II/III obesity

**Figure S3:** In-hospital mortality among patients with normal BMI as compared with obesity (all classes)


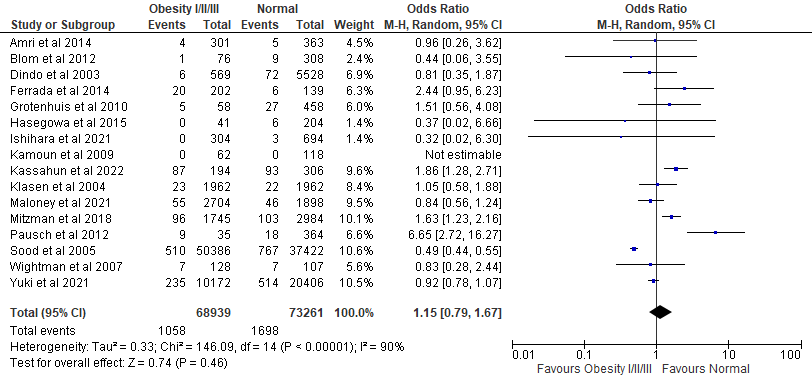

Supplement: zrad026_Supplementary_Data [file zrad026_supplementary_data.zip › Supplementary_Material.docx]
